# Enzyme-Assisted Ultrasonic Extraction and Antioxidant Activities of Polysaccharides from *Schizochytrium limacinum* Meal

**DOI:** 10.3390/foods13060880

**Published:** 2024-03-14

**Authors:** Nuohan Zhang, Wenwei Chen, Xinyu Li, Xinmiao Chen, Yuchen Wang, Guangrong Huang, Jiaxian Wang, Zhenbao Jia

**Affiliations:** 1 College of Life Sciences, China Jiliang University, Hangzhou 310018, China; nuohansephire@gmail.com (N.Z.); 15990179129@163.com (X.L.); cxmdyx2001@163.com (X.C.); careywangyc@163.com (Y.W.); 07b0904052@cjlu.edu.cn (G.H.); 18868432919@163.com (J.W.); zhenbaojia@cjlu.edu.cn (Z.J.); 2 Key Laboratory of Specialty Agri-Product Quality and Hazard Controlling Technology of Zhejiang Province, Hangzhou 310018, China

**Keywords:** marine microalgae, polysaccharide, enzyme-assisted ultrasonic extraction, antioxidant activity

## Abstract

Enzyme-assisted ultrasonic extraction (EAUE) was utilized and optimized for extracting polysaccharides from *Schizochytrium limacinum* meal (SLMPs) via the response surface methodology. The optimal EAUE conditions were determined as follows: enzyme concentration at 5.18%, ultrasonic temperature at 53 °C, ultrasonic duration of 40 min, ultrasonic power at 60 W, and a liquid-to-material ratio of 34 mL/g, achieving a polysaccharide extraction yield of 11.86 ± 0.61%. The purified polysaccharide component, SLMP1-1, isolated using DEAE Sepharose Fast Flow and Sephadex G-100 columns, exhibited potent antioxidant activity. SLMP1-1, with a molecular weight of 25.5 kDa, comprises glucose, mannose, arabinose, and galactose in a molar ratio of 16.39:14.75:1:693.03. ^1^H NMR analysis revealed the α configuration of SLMP1-1. Antioxidant assessments, including DPPH, ABTS, and ferric ion reduction assays, were detected with inhibitory values at 21.82–82.98%, 38.21–98.46%, and 3.30–20.30% at 0.2–1.0 mg/mL. This confirmed the effective antioxidant capacity of SLMP1-1, which is notably enhanced post oral and gastric digestion. The findings suggest that polysaccharides extracted from *Schizochytrium limacinum* meal hold significant promise as natural antioxidants.

## 1. Introduction

*Schizochytrium limacinum,* a heterotrophic marine alga, serves as a significant source of DHA algal oil [1], bioenergy preparation [2], and feed preparation [3]. Its growth characteristics determine that the alga is suitable for large-scale artificial cultivation. *Schizochytrium limacinum* meal, produced by extracting DHA, makes up over 64% of the total mass of algae, and most of these meals are discarded rather than being used as feed or fertilizer, resulting in resource wastage [4]. The algae meal contains many active substances, including polysaccharides and proteins. And numerous studies have shown that polysaccharides exhibit various physiological functions, such as anti-inflammatory [5], neuroprotective [6], anti-diabetic [7], anti-tumor [8], and antioxidant activities [9]. However, most current studies on *Schizochytrium limacinum* focus on the DHA algal oil, and there are limited studies on the polysaccharides from *Schizochytrium limacinum* meal. Moreover, the relevant research is relatively old, and the techniques are traditional. Therefore, studying the extraction and activity of polysaccharides from *Schizochytrium limacinum* meal holds great significance.

Traditional polysaccharide extraction methods like hot water extraction are inefficient and can degrade the active ingredients of the polysaccharides [10,11,12]. To overcome these limitations, new techniques such as enzyme extraction [13], ultrasonic extraction [14], microwave extraction [15], and supercritical extraction [16] have been explored. Enzyme-assisted ultrasonic extraction (EAUE) is a novel technique that combines enzymatic and ultrasonic methods, offering high yields and the preservation of the active ingredients of the polysaccharides. This results from the enzyme promoting the separation of polysaccharides by degrading biological barriers and altering cell permeability [17]. Ultrasound’s ability to induce cavitation, heat, and mechanical effects is the foundation of ultrasound-assisted extraction, and cavitation is the most crucial element [18]. When high-frequency ultrasonic vibration generates a cavitation effect on the cell wall, a few weak tension areas will be torn into tiny cavities, and the fluid will be released into the solvent. The rapid expansion and closure of these microscopic cavities results in a violent collision of liquid particles, creating a mild-temperature environment that promotes ingredient solubility and separation [19,20].

As a result, this study targeted optimizing the enzyme-assisted ultrasonic extraction (EAUE) method of *Schizochytrium limacinum* meal polysaccharides (SLMPs). The response surface method and Box–Behnken design were used to analyze the impact of five factors on the extraction yield of polysaccharides, including enzyme concentration, ultrasonic temperature, ultrasonic time, and the liquid-to-material ratio. After column separation and purification, pure polysaccharides were prepared for further structural analysis, such as infrared spectra and ^1^H NMR spectroscopic analysis. The molecular weight and monosaccharide composition of SLMPs were determined through the HPGPC and HPLC methods, respectively. The study also evaluates the antioxidant activity and simulated digestion of SLMPs in the mouth, intestine, and stomach through DPPH, ABTS radical scavenging, and ferric ion reduction ability assays for the development of *Schizochytrium limacinum* meal.

## 2. Materials and Methods

### 2.1. Materials and Equipment

The meal from *Schizochytrium limacinum*, obtained after DHA algal oil extraction by Lvqi Biotechnology Company, Wuxi, China, was cleaned, dried at 55 °C, and ground into powder. Papain (pH 6.0; 800,000 U/g) from Xuemei Enzyme Preparation Technology in Wuxi, China, was utilized for hydrolysis reaction. DEAE Sepharose Fast Flow and Sephadex G-100 column cellulose were supplied by Amersham Biosciences. The dialysis bags (MwCO 8000–14,000 D) were provided by Shanghai Rongpeng Information Technology Co., Ltd., Shanghai, China.

The ultrasonic cleaner (KQ2200DE) was provided by Kunshan Shumei Company, Kunshan, China, and was barrel-type. A high-speed micro centrifuge (Type CF 15RN) was provided by Himac, a company from Japan.

### 2.2. Enzyme-Assisted Ultrasonic Extraction of Polysaccharides (SLMPs)

Polysaccharides were extracted under these specified conditions: A 1000-mL glass beaker was filled with *Schizochytrium limacinum* powder (10 g), a particular concentration of papain (2, 3, 4, 5, and 6%), and a phosphate buffer (0.2 mol/L) in liquid-to-material ratios (15, 20, 25, 30, and 35 mL/g). Then, SLMPs were extracted using various ultrasonic times (10, 20, 30, 40, and 50 min), ultrasonic powers (40, 50, 60, 70, and 80 W), and ultrasonic temperatures (30, 40, 50, 60, and 70 °C) at a 40 KHz ultrasonic frequency. 

After extraction, the mixture was centrifuged for 15 min at 12,000 rpm, and the residue was removed. The supernatant was precipitated with anhydrous ethanol for 24 h below 4 °C. Free proteins were eliminated through the Sevag method [21] and washed three times using acetone and anhydrous ethanol ahead of sediment collection. After using a dialysis bag with MWCO of 14,000 Da to eliminate materials with low molecular weight, the SLMPs were collected by lyophilizing the crude polysaccharides.

The phenol–sulfuric acid method was used to measure the SLMPs concentration [22]. And the yield of SLMPs was calculated using the following formula, which is based on dry weight.

(1) $$\mathrm{Yield}\;\left(\%\right)=\frac{\mathrm{the}\;\mathrm{dry}\;\mathrm{weight}\;\mathrm{of}\;\mathrm{SLMPs}}{\mathrm{the}\;\mathrm{weight}\;\mathrm{of}\;Schizochytrium\;Limacinum\;\mathrm{meal}}$$

### 2.3. Experimental Design

On the basis of a single-factor experiment, the yield of SLMPs was optimized by the response surface method and Box–Behnken design (BBD) [23]. 

According to BBD, the yield of the SLMPs was the response value, and the independent variables were ultrasonic time, ultrasonic temperature, enzyme concentration, ultrasonic power, and liquid-to-material ratio, which were based on the results of the single-factor experiment. The yield of the polysaccharides from the algal residue was stated as the second-order polynomial:

(2) $$Y=\alpha_{0}+\sum \alpha_{m}X_{m}+\sum \alpha_{\mathrm{mm}}X_{m}^{2}+\sum \sum \alpha_{\mathrm{mn}}X_{m}X_{n}$$

where Y is the predicted yield of SLMPs, α_0_ is a constant, α_m_ is a linear factor, α_mm_ is a quadratic factor, α_mn_ is an interactive term factor, and X_m_ and X_n_ are the independent variables representing four arguments.

### 2.4. Separation and Purification

To purify the SLMPs, 100 mg SLMPs were dissolved in 20 mL of deionized water and then processed through the DEAE Sepharose Fast Flow column (2.5 cm × 30 cm). At a flow rate of 0.5 mL/min, the column was eluted with solutions of varying NaCl concentrations (0.1, 0.2, 0.3, 0.4, and 0.5 mol/L) to obtain the fractions with the highest antioxidant activity. Deionized water was used for separation and purification using a Sephadex G-100 column (2.6 cm × 60 cm) at 0.5 mL/min. Then, the components with the greatest antioxidant activity were collected and freeze-dried for subsequent use.

### 2.5. In Vitro Simulated Saliva-Gastrointestinal Digestion of SLMP1-1

The reported approach of Wang et al. [24] was applied to assess the in vitro simulated digestion experiment of SLMP1-1 with a few modifications. SLMP1-1 solutions of various concentrations were digested sequentially in simulated saliva, gastric juice, and intestinal fluid, with the digested solutions prepared for analysis.

Solutions of SLMP1-1 at various concentrations (0.1, 0.2, 0.3, 0.4, and 0.5 mg/mL) were prepared. Then, 100 mL of the sample liquid and simulated saliva were placed in triangular bottles and stirred with a magnetic agitator. After 1 h, 10 mL of the reaction solution was removed, rapidly placed in boiling water for 5 min, and centrifuged for 15 min at 4500 rpm. The supernatant was retained for oral digestion. Deionized water was used as a blank control. In a conical flask, 10 mL of the orally digested solution were placed. Gastric juice was added, digested for 1.5 h at 37 °C, and then placed in boiling water for 20 min to terminate digestion. The mixture was centrifuged for 15 min at 4500 rpm, and the supernatant was retained for gastric digestion. Then, 10 mL of the gastrically digested solution was added to the intestinal fluid. The digestive fluid was collected after 2.5 h and centrifuged for 15 min at 4500 rpm. The supernatant was collected as intestinal digestion.

### 2.6. Analysis of SLMP1-1

#### 2.6.1. Measurement of Molecular Weight of SLMP1-1

The molecular weight of SLMP1-1 was determined through high-performance gel permeation chromatography (HPGPC). 2 mg of SLMP1-1 powder was dissolved in 1 mL of a 0.02 M potassium dihydrogen phosphate solution before passing through a 0.22 μm organic filter membrane. An Agilent G-5000 PWXL (7.8 × 300 mm i.d., 10 μm) and a G-3000 PWXL (7.8 × 300 mm i.d., 5 μm) in-series gel columns were used to elute 20 μL of the SLMP1-1 solution at a flow rate of 0.6 mL/min, with 0.02 M potassium dihydrogen phosphate solution as the mobile phase.

The known molecular weight of Pullulan series glucan (6000–2,500,000 Da) was prepared in a 1 mg/mL standard solution and then analyzed by HPGPC under the same conditions. The standard curve was constructed with elution time as the abscissa and the log of molecular weight as the ordinate to calculate the molecular weight of SLMP1-1.

#### 2.6.2. Monosaccharide Composition of SLMP1-1

The monosaccharide composition was determined by the high-performance liquid chromatography (HPLC) method, according to the study of Y. Chen et al. [25]. Trifluoroacetic acid (TFA, 10.0 mL, 2.0 mol/L) was combined with the SLMP1-1 sample (10.0 mg) in a sealed tube with N_2_ filled and hydrolyzed at 110 °C for 6 h. The hydrolyzed sample was re-extracted with methanol, and then the extract was dried by N_2_ to eliminate TFA. In 0.3M NaOH (100 μL), the monosaccharide standard solution and polysaccharide hydrolysate were dissolved before mixing them with 120 μL of 0.5 M PMP methanol liquor. In a water bath, the combination was heated to 70 °C for 2 h. 

Then, 800 μL of methylene chloride was added to the compounds to eliminate PMP through three separate extractions after being warmed to room temperature and neutralized with 100 μL of 0.3 M HCl. Following a 0.45 μm membrane filtering step, the aqueous layer was subjected to HPLC analysis with DAD (Agilent 1100). Chromatographic data were recorded, and monosaccharides in the sample were identified and quantified through comparative analysis with known standards. A C18 column (5 μm, 4.6 mm × 250 mm) was used in the column oven, which was adjusted to 30 °C. With a detection wavelength of 250 nm and an injection volume of 5 μL, a flow rate of 1.5 mL/min was used for the mobile phase of acetonitrile and sodium phosphate buffer (pH 6.6, 0.1 M).

#### 2.6.3. Infrared and ^1^H NMR Spectroscopy

After freeze drying, the polysaccharide sample SLMP1-1 was mixed with KBr. The IR spectra were obtained by infrared spectroscopy scanning [26]. Then, 20 mg of SLMP1-1 was dissolved in D_2_O (3.0 mL, 99.8%), and the mixture was then placed in a 5 mm NMR tube. A Bruker Avance 400 MHz NMR spectrometer was used to obtain the ^1^H NMR spectra at 20 °C [27].

### 2.7. Antioxidant Activity of SLMP1-1

The DPPH, ABTS radical scavenging activity, and ferric reducing power of SLMP1-1 were measured according to Abbou et al. [28]. The antioxidant activity of SLMP1-1 was detected, and digestion products were simulated separately. The antioxidant activity of vitamin C (Vc) was measured as a positive control.

### 2.8. Statistical Analysis

Every experiment was conducted three times. The response surface (RSM) and the Box–Behnken design (BBD) were analyzed using Design-Expert software (Stat-Ease, 218 US, version 10.0).

## 3. Results and Discussion

### 3.1. The Fitted Model and Response Surface Analysis

The extraction conditions of the SLMPs were optimized using the Box–Behnken design (BBD). The enzyme concentration (A), ultrasonic temperature (B), ultrasonic time (C), liquid-to-material ratio (D), and ultrasonic power (E) were evaluated as five independent variables. The outcomes are reported in Table 1. The yield (%) was calculated according to the Formula (1).

In the second-order polynomial regressive equation for the EAUE, the yield of the SLMPs (%) served as the response value, and the result was as follows:

Y = 11.79 + 0.55A + 0.49B − 0.43C + 0.53D + 0.36E + 0.34AB + 0.33AC + 0.12AD + 0.24AE + 0.47BC + 1.08BD − 0.09BE + 0.45CD + 0.52CE − 0.54DE − 2.03A^2^ − 1.55B^2^ − 1.11C^2^ − 1.12D^2^ − 1.07E^2^ (3)

Table 1 indicates that both the *p*-value of the fitted model (less than 0.0001) and the lack-of-fit value were highly significant, suggesting the model’s accuracy in predicting the yield of the extraction process. The fitting model accounted for 98.64% of the observed variations and clarified 97.56% of all variations, according to the adjusted determination (A_dj_.R^2^) and determination coefficient (R^2^) coefficients of 0.9864 and 0.9756, respectively. The experiment was highly accurate and reliable, supported by the low C.V.% (2.17) of the data.

**Figure 1:**
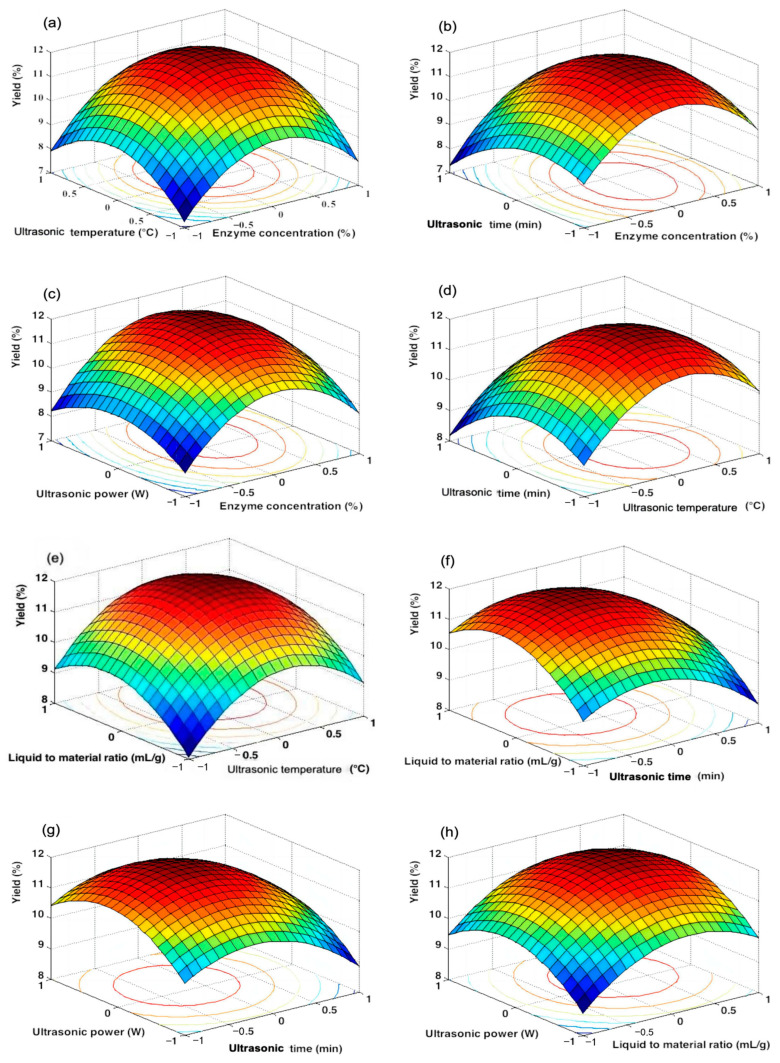
Response surface plots for the SLMPs yield. (**a**) Effect of extraction temperature and enzyme concentration on the SLMPs yield; (**b**) Effect of extraction time and enzyme concentration on the SLMPs yield; (**c**) Effect of ultrasonic power and enzyme concentration on the SLMPs yield; (**d**) Effect of extraction time and extraction temperature on the SLMPs yield; (**e**) Effect of liquid-to-material ratio and extraction temperature on the SLMPs yield; (**f**) Effect of liquid-to-material ratio and extraction time on the SLMPs yield; (**g**) Effect of ultrasonic power and extraction time on the SLMPs yield; and (**h**) Effect of ultrasonic power and liquid-to-material ratio on the SLMPs yield.

Table 2 shows that the linear coefficients (A, B, C, D, and E), interaction coefficients (AB, AC, AE, BC, BD, CD, CE, and DE), quadratic term coefficient (A^2^, B^2^, C^2^, D^2^, and E^2^), and the interaction coefficient of AD and BE were significant. The interactions of AD and BE had a minimal impact on the yield of the SLMPs. Figure 1 reveals the effect of various factors and their interactions on the yield of the polysaccharides.

Among them, the response surfaces (Figure 1e–h) showed the most significant changes. The yield of the SLMPs increased with the increase in liquid-to-material ratio, reaching its maximum value at an ultrasonic power of 62 W and an ultrasonic temperature of 53 °C. This trend suggests that higher temperatures and solvent concentrations enhance enzyme activity and catalysis and make the irregular movement of polysaccharides obvious, thereby increasing solvent diffusion into the components and boosting extraction efficiency [29]. 

In addition, as shown in Figure 1d–g, the trend of ultrasonic power from 50 to 70 W and ultrasonic temperature from 40 to 60 °C indicated an increase in the yield of the SLMPs. This can be attributed to the effects of ultrasound, particularly the cavitation effect. The cavitation effect, associated with increases in temperature and pressure, contributes to the yield of the SLMPs through the formation of micro-streams, turbulent phenomena, and the rupture of small-sized droplets under high-intensity ultrasound [30,31].

### 3.2. Optimization of the Extraction of SLMPs

According to the RSM (response surface methodology) analysis, the optimal circumstances for the SLMPs extraction were as follows: 5.18% enzyme concentration, 53 °C ultrasonic temperature, 40 min ultrasonic time, 60 W ultrasonic power, 34 mL/g liquid-to-material ratio. The polysaccharide extraction yield was 11.86 ± 0.61%. Taking into account the practicality of the operation, these optimal conditions were adjusted, and the experiment was repeated three times to obtain actual experimental data. A relative deviation of 1.41% between the predicted value (12.03%) and the actual yield values confirmed the model’s efficacy in capturing the relationship between the variables and response values.

### 3.3. Purification of SLMPs

The extracted crude SLMPs were loaded on the DEAE-Sepharose Fast Flow column with ion exchange chromatography, and the eluent was collected. When the polysaccharide components were adsorbed on the ion exchange column, polysaccharides with different charge properties carried by the eluent flowing through the ion exchange column were successively eluted.

**Figure 2:**
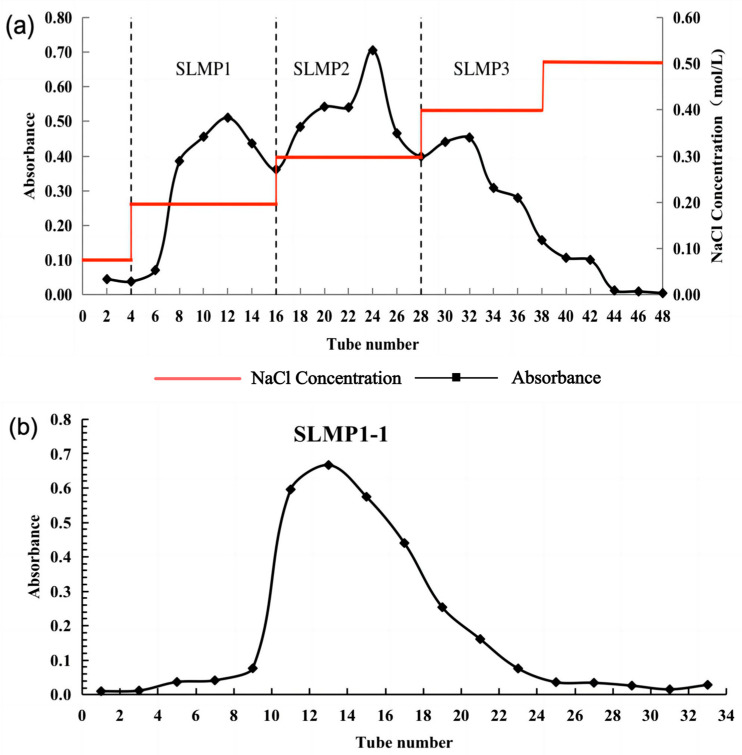
Purification of the polysaccharides. (**a**) DEAE-Sepharose Fast Flow column gel filtration of SLMPs; (**b**) Sephadex G-100 gel filtration of SLMP1 (the absorbance is at 517 nm).

As shown in Figure 2a, the polysaccharide content of SLMP1, SLMP2, and SLMP3 accounts for 31.50%, 42.89%, and 25.61% of the total saccharide content, respectively. The measurement of the DPPH antioxidant activity of these three separated polysaccharide components revealed that SLMP1 exhibited the highest antioxidant activity among them. Subsequently, deionized water was used to load SLMP1 into the Sepharose CL-100 column. A single peak, designated as SLMP1-1, was obtained (see Figure 2b) and collected via freeze-drying.

### 3.4. Composition and Structure Analysis

**Figure 3:**
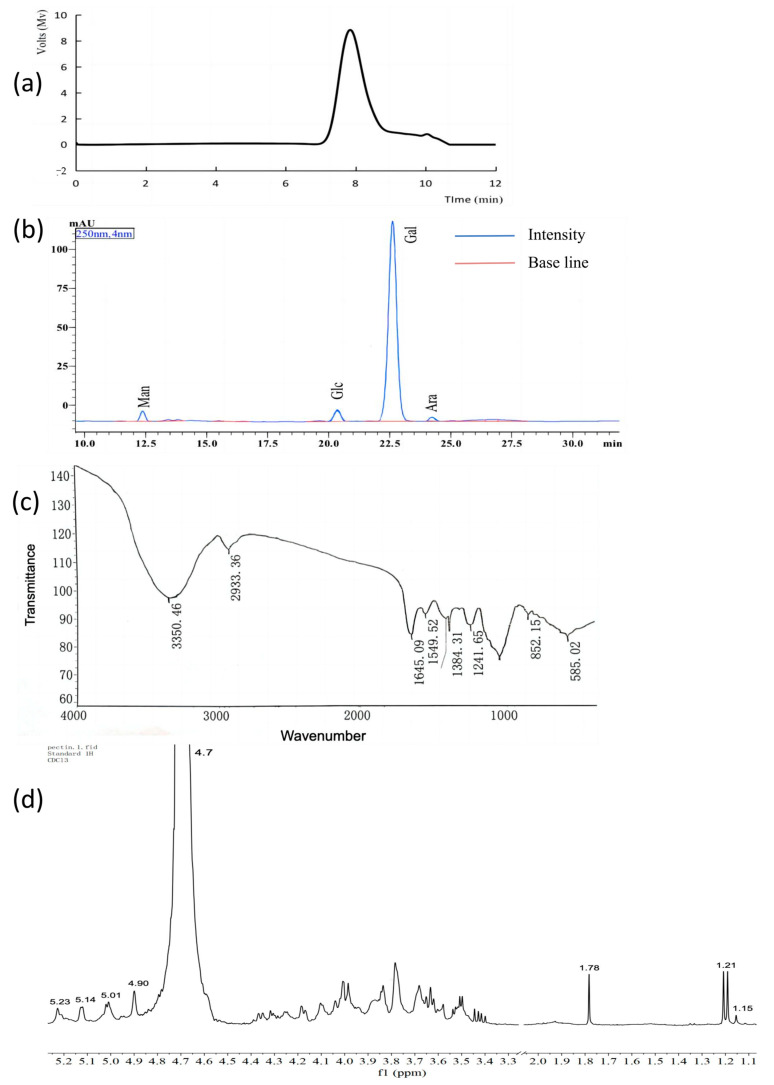
(**a**) Molecular weights of SLMP1-1; (**b**) Chromatogram of the PMP derivation of SLMP1-1; (**c**) FT-IR spectra of SLMP1-1; (**d**) NMR spectral analysis of SLMP1-1.

The molecular weight of SLMP1-1, determined by HPGPC, was 25.5 kDa, as shown in Figure 3a. A single, symmetric peak occurred at 8 min. According to the HPLC analysis (see Figure 3b), SLMP1-1 primarily consisted of glucose, mannose, arabinose, and galactose, with a molar ratio of 16.39:14.75:1:693.03, respectively. Notably, the proportion of galactose—which remains the main composition of SLMPs extracted by EAUE—was higher compared to the traditional hot water extraction. Both methods yielded small amounts of mannose (Man) and glucosamine (GlcN).

The Fourier-transform infrared spectrum of SLMP1 (Figure 3c) revealed a characteristic saccharide absorption peak in the range of 400–4000 cm^−1^. A broad absorption peak at 3350 cm^−1^ was attributed to intramolecular or intermolecular O-H hydrogen bond stretching vibration. The methyl and methylene C-H bond stretching vibration peaks appeared at 2933 cm^−1^, interacting within the interaction of the carbon chain [32,33]. Strong absorption peaks were observed at both 1241 cm^−1^ and 1645 cm^−1^, respectively, corresponding to the stretching vibration of -O-SO_3_-H- and the C=O stretching vibration of the amide -HN-C=OR [34]. A weak absorption peak from the O-H variable angle vibration appeared at 1549 cm^−1^. SLMP1 also showed a weak absorption peak for the C-H variable angular vibration at 1384 cm^−1^ due to the presence of uronic acid [35]. The absorption peak at approximately 1000 cm^−1^ corresponds to the hydroxyl group of the pyran ring [36], and the characteristic absorption peak of α-pyranose was observed at 852 cm^−1^ [37]. These are typical features of polysaccharides [38].

Nuclear magnetic resonance (NMR) provides structural information on the polysaccharides, including the identification of α-monosaccharide or β-clarification of heterogeneous configurations, as well as the establishment of linkage patterns [39]. Typically, the coupling constant for the anomeric protons in α-configuration pyranosides shifts around δ4.8–5.8, while for β-configuration pyranosides, it is around δ4.4–4.8. The ^1^H NMR results for SLMP1-1, shown in Figure 3d, reveal chemical shifts for isomeric protons between δ4.90 and 5.23 ppm, suggesting an α configuration. The prominent signal at δ 4.7 originates from the D_2_O solvent, and signals at δ1.15–1.21 ppm and δ1.78 ppm correspond to -CH_3_ and -CH groups, respectively, aligning with the characteristic absorption at 2933 cm^−1^ in the FTIR spectrum [40,41]. The presence of four signals (δ4.90, 5.01, 5.14, 5.23 ppm) in the anomeric proton region (δ4.9–5.3 ppm) indicates that SLMP1-1 likely contains four types of monosaccharide residues, corresponding to the chemical shifts for α-Gal, α-Glc, α-Man, and α-Ara, respectively. And the range δ3.3–4.4 indicates combined proton signals from H-2 to H-6 in the glycosidic bond [42,43,44]. These observations are in agreement with the FT-IR results and the monosaccharide composition analysis.

### 3.5. Study on Antioxidant Activity

The antioxidant activity of a substance is influenced by various factors, and relying solely on one antioxidant test may not adequately reflect its antioxidant capabilities. The antioxidant capacity of the polysaccharides is related to their ability to chelate metal ions, scavenge free radicals, and donate electrons. 

**Figure 4:**
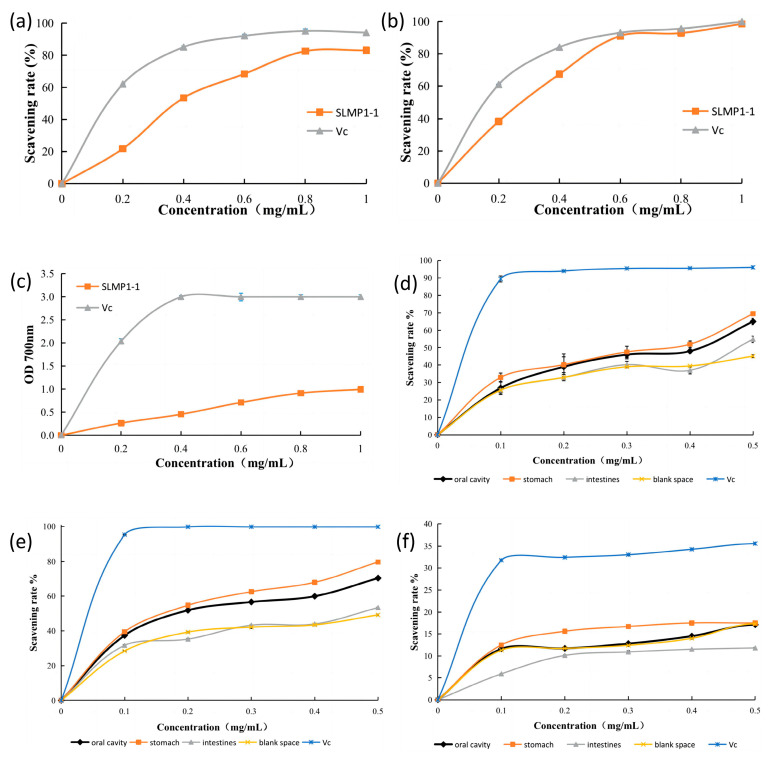
Antioxidant activity of SLMP1-1. (**a**) DPPH radical scavenging activity; (**b**) ABTS radical scavenging activity; (**c**) Ferric-reducing antioxidant power; (**d**) DPPH radicals scavenging activity of digested fractions; (**e**) ABTS radicals scavenging activity of digested fractions; (**f**) Ferric reducing power of digested fractions; values are means ± SD (n = 3); and the antioxidant activity of vitamin C (Vc) is the positive control.

The DPPH free radical is widely employed to assess a compound’s ability to scavenge free radicals. The dose-effect relationship between the concentration of SLMP1-1 and the rate of DPPH scavenging is shown in Figure 4a, and the IC_50_ value is 0.39 mg/mL. The inhibitory trend of Vc on DPPH free radical activity is similar to that of SLMP1-1. According to Hosseinpouri, Mohammadi, Ehsandoost, Sharafi-Badr, and Obeidi [45], SLMP1-1 exhibits significantly stronger antioxidant activity than polysaccharides from *Sargassum* (FSA), which have a DPPH scavenging activity with an IC_50_ value of 0.157 mg/mL. 

The ABTS method is another commonly used indicator for estimating total antioxidant activity. The trend in ABTS free-radical scavenging activity is consistent with the DPPH trend and shows a high concentration dependence, as depicted in Figure 4b. According to Shao, Chen, and Sun [46], at a concentration of 1.0 mg/mL, the radical scavenging rates of the polysaccharides from *Sargassum horneri* reached nearly 100%, similar to those of SLMP1-1. 

Antioxidants eliminate free radicals by donating electrons, and the stronger their reducing ability, the more potent their antioxidant activity. Figure 4c reveals that the ferric ion reduction ability of SLMP1-1 increases with concentration. Additionally, SLMP1-1 has a higher OD_700nm_ value (0.99, 1 mg/mL), indicating a stronger total antioxidant activity compared to different polysaccharide components isolated from *S. horneri* (0.47–0.69, 1 mg/mL) [47].

As shown in Figure 4d–f, SLMP1-1 exhibits increased DPPH scavenging activities after undergoing salivary and gastric digestion. This may be due to the action of enzymes like α-amylase and pepsin in gastric juice, which break glycosidic bonds in polysaccharides, thereby quickly releasing more antioxidant substances and lower-molecular-weight polysaccharides [48].

However, after intestinal digestion, the DPPH scavenging activity of SLMP1-1 decreased significantly, with a 7% reduction observed. One possible explanation is that the increased pH value in intestinal fluids leads to the hydrolysis of SLMP1-1, thereby increasing the polarity of the polysaccharides and making it more difficult to induce free radical reactions. Like the DPPH findings, ABTS results also indicated that scavenging activity significantly increased after gastric digestion but decreased after intestinal digestion. Upon simulating in vivo digestion, no significant change was found in the total reducing power of SLMP1-1, which aligns with the findings of Schmelzer, Schöps, Reynell, Ulbrich-Hofmann, Neubert, and Raith [49].

## 4. Conclusions

In this study, the extraction conditions for polysaccharides from the algae (*Schizochytrium limacinum*) were optimized using a response surface methodology (RSM). The optimal process parameters for enzyme-assisted ultrasonic extraction (EAUE) were identified as follows: a 5.18% enzyme concentration, an ultrasonic temperature of 53 °C, an ultrasonic time of 40 min, an ultrasonic power of 60 W, a liquid-to-material ratio of 34 mL/g. The polysaccharide extraction yield was 11.86%.

The extracted crude polysaccharides were further separated and purified using a DEAE-Sepharose Fast Flow column and Sephadex G-100 columns. Among the separated components, SLMP1-1 was identified to possess the highest antioxidant activity, showing significant capabilities in DPPH free radical scavenging, ABTS radical scavenging, and ferric-reducing antioxidant power. In simulated in vivo conditions, its antioxidant activities generally increased after gastric digestion but decreased following intestinal digestion. 

Additional analyses included Fourier-transform infrared (FT-IR) spectroscopy, which confirmed the characteristic absorption peaks associated with the polysaccharides in SLMP1. HPGPC determined the molecular weight of SLMP1-1 to be 25.5 kDa. Furthermore, HPLC analysis revealed that SLMP1-1 primarily consisted of glucose, mannose, galactose, and arabinose at a molar ratio of 16.39:14.75:1:693.03. ^1^H NMR spectroscopy verified that SLMP1-1 has an α configuration. Overall, these findings serve as a valuable reference for enhancing the processing and utilization of *Schizochytrium limacinum* residues. Further research is recommended to explore the relationship between the molecular structure of these polysaccharides and their antioxidant activity.

## Figures and Tables

**Table 1:** Box–Behnken design and response values for the yield of SLMPs. The yield (%) is the proportion of the dry weight of SLMPs to the weight of *Schizochytrium Limacinum* meal.

Number	A (Enzyme Concentration, %)	B (Ultrasonic Temperature, °C)	C (Ultrasonic Time, min)	D (Liquid to Material Ratio, mL/g)	E (Ultrasonic Power, W)	Yield (%)
1	−1 (4)	−1 (40)	0 (40)	0 (30)	0 (60)	7.92
2	1 (6)	−1 (40)	0 (40)	0 (30)	0 (60)	8.01
3	−1 (4)	1 (60)	0 (40)	0 (30)	0 (60)	7.96
4	1 (6)	1 (60)	0 (40)	0 (30)	0 (60)	9.42
5	0 (5)	0 (50)	−1 (30)	−1 (20)	0 (60)	9.65
6	0 (5)	0 (50)	1 (50)	−1 (20)	0 (60)	8.06
7	0 (5)	0 (50)	−1 (30)	1 (40)	0 (60)	10.13
8	0 (5)	0 (50)	1 (50)	1 (40)	0 (60)	10.35
9	0 (5)	−1 (40)	0 (40)	0 (30)	−1 (50)	8.16
10	0 (5)	1 (60)	0 (40)	0 (30)	−1 (50)	9.46
11	0 (5)	−1 (40)	0 (40)	0 (30)	1 (70)	9.10
12	0 (5)	1 (60)	0 (40)	0 (30)	1 (70)	10.05
13	−1 (4)	0 (50)	−1 (30)	0 (30)	0 (60)	9.04
14	1 (6)	0 (50)	−1 (30)	0 (30)	0 (60)	9.57
15	−1 (4)	0 (50)	1 (50)	0 (30)	0 (60)	7.16
16	1 (6)	0 (50)	1 (50)	0 (30)	0 (60)	9.02
17	0 (5)	0 (50)	0 (40)	−1 (20)	−1 (50)	8.21
18	0 (5)	0 (50)	0 (40)	1 (40)	−1 (50)	10.29
19	0 (5)	0 (50)	0 (40)	−1 (20)	1 (70)	10.16
20	0 (5)	0 (50)	0 (40)	1 (40)	1 (70)	10.09
21	0 (5)	−1 (40)	−1 (30)	0 (30)	0 (60)	9.42
22	0 (5)	1 (60)	−1 (30)	0 (30)	0 (60)	9.59
23	0 (5)	−1 (40)	1 (50)	0 (30)	0 (60)	7.58
24	0 (5)	1 (60)	1 (50)	0 (30)	0 (60)	9.63
25	−1 (4)	0 (50)	0 (40)	−1 (20)	0 (60)	7.67
26	1 (6)	0 (50)	0 (40)	−1 (20)	0 (60)	8.58
27	−1 (4)	0 (50)	0 (40)	1 (40)	0 (60)	8.47
28	1 (6)	0 (50)	0 (40)	1 (40)	0 (60)	9.87
29	0 (5)	0 (50)	−1 (30)	0 (30)	−1 (50)	10.26
30	0 (5)	0 (50)	1 (50)	0 (30)	−1 (50)	8.57
31	0 (5)	0 (50)	−1 (30)	0 (30)	1 (70)	9.72
32	0 (5)	0 (50)	1 (50)	0 (30)	1 (70)	10.09
33	−1 (4)	0 (50)	0 (40)	0 (30)	−1 (50)	7.76
34	1 (6)	0 (50)	0 (40)	0 (30)	−1 (50)	8.52
35	−1 (4)	0 (50)	0 (40)	0 (30)	1 (70)	8.05
36	1 (6)	0 (50)	0 (40)	0 (30)	1 (70)	9.77
37	0 (5)	−1 (40)	0 (40)	−1 (20)	0 (60)	9.27
38	0 (5)	1 (60)	0 (40)	−1 (20)	0 (60)	8.06
39	0 (5)	−1 (40)	0 (40)	1 (40)	0 (60)	7.87
40	0 (5)	1 (60)	0 (40)	1 (40)	0 (60)	10.99
41	0 (5)	0 (50)	0 (40)	0 (30)	0 (60)	11.68
42	0 (5)	0 (50)	0 (40)	0 (30)	0 (60)	11.79
43	0 (5)	0 (50)	0 (40)	0 (30)	0 (60)	11.66
44	0 (5)	0 (50)	0 (40)	0 (30)	0 (60)	11.77
45	0 (5)	0 (50)	0 (40)	0 (30)	0 (60)	11.96
46	0 (5)	0 (50)	0 (40)	0 (30)	0 (60)	11.87

**Table 2:** Analysis of variance of predicted model.

Source	Sum of Squares	DF	*F*-Value	*p*-Value
Model	75.65	20	90.93	<0.0001
A	4.75	1	114.25	<0.0001
B	3.83	1	92.12	<0.0001
C	2.98	1	71.75	<0.0001
D	4.41	1	106.02	<0.0001
E	2.10	1	50.55	<0.0001
AB	0.47	1	11.28	0.0025
AC	0.44	1	10.47	0.0034
AD	0.06	1	1.44	0.2409
AE	0.23	1	5.54	0.0268
BC	0.88	1	21.24	<0.0001
BD	4.69	1	112.69	<0.0001
BE	0.03	1	0.74	0.3990
CD	0.82	1	19.69	<0.0001
CE	1.06	1	25.51	<0.0001
DE	1.16	1	27.78	<0.0001
A^2^	35.88	1	862.50	<0.0001
B^2^	21.06	1	506.25	<0.0001
C^2^	10.69	1	256.96	<0.0001
D^2^	10.90	1	262.02	<0.0001
E^2^	10.04	1	241.34	<0.0001
Residual	1.04	25		
Lack of Fit	0.9717	20	3.56	0.0818
Pure Error	0.0682	5		
Total	76.69	45		
C.V.%	2.17			
R^2^	0.9864			
Adj-R^2^	0.9756			

## Data Availability

The original contributions presented in the study are included in the article, further inquiries can be directed to the corresponding author.
